# Harmony in the Molecular Orchestra of Hearing: Developmental Mechanisms from the Ear to the Brain

**DOI:** 10.1146/annurev-neuro-081423-093942

**Published:** 2024-07-01

**Authors:** Sonja J. Pyott, Gabriela Pavlinkova, Ebenezer N. Yamoah, Bernd Fritzsch

**Affiliations:** 1 Department of Otorhinolaryngology and Head and Neck Surgery, University Medical Center Groningen, Graduate School of Medical Sciences, and Research School of Behavioral and Cognitive Neurosciences, University of Groningen, Groningen, The Netherlands; 2 Laboratory of Molecular Pathogenetics, Institute of Biotechnology, Czech Academy of Sciences, Vestec, Czechia; 3 Department of Physiology and Cell Biology, School of Medicine, University of Nevada, Reno, Nevada, USA; 4 Department of Neurological Sciences, University of Nebraska Medical Center, Omaha, Nebraska, USA

**Keywords:** cochlea, cochlear nucleus, superior olivary complex, inferior colliculus, medial geniculate body, auditory cortex

## Abstract

Auditory processing in mammals begins in the peripheral inner ear and extends to the auditory cortex. Sound is transduced from mechanical stimuli into electrochemical signals of hair cells, which relay auditory information via the primary auditory neurons to cochlear nuclei. Information is subsequently processed in the superior olivary complex, lateral lemniscus, and inferior colliculus and projects to the auditory cortex via the medial geniculate body in the thalamus. Recent advances have provided valuable insights into the development and functioning of auditory structures, complementing our understanding of the physiological mechanisms underlying auditory processing. This comprehensive review explores the genetic mechanisms required for auditory system development from the peripheral cochlea to the auditory cortex. We highlight transcription factors and other genes with key recurring and interacting roles in guiding auditory system development and organization. Understanding these gene regulatory networks holds promise for developing novel therapeutic strategies for hearing disorders, benefiting millions globally.

## INTRODUCTION

The sense of hearing enables communication, spatial awareness, speech and language use, and the appreciation of music and complex soundscapes. The auditory pathway spans from the cochlea, the sensory organ in the inner ear, to various auditory structures within the brainstem and eventually to higher auditory processing centers. This intricate network of ascending, descending, and reciprocal connections is responsible for the encoding, transmitting, and interpreting auditory information.

Recent advances have provided valuable insights into the molecular mechanisms that govern auditory system development and that are essential for the proper functioning of the individual auditory structures as well as their connectivity (Fritzsch et al. 2022, Molnár et al. 2020, Rolls et al. 2023). These insights complement our understanding of the physiological mechanisms underlying auditory processing and are vital to developing novel therapeutic strategies to treat the various hearing disorders that affect millions worldwide (Elliott et al. 2022).

In this comprehensive review, we employ an integrative perspective, outlining genetic and molecular insights into the developmental organization of key auditory structures in the periphery, the brainstem and midbrain, and the thalamus and cortex, the three major sections in the molecular orchestra of mammalian hearing. We emphasize areas of mechanistic overlap, highlight new methodological tools and approaches, and prioritize areas for future research. We also refer readers to other references for more detailed insight into the physiology of the auditory pathways.

## AUDITORY PERIPHERY: MAKING AND DIVERSIFYING THE SENSORY HAIR CELLS AND PRIMARY AUDITORY NEURONS

The initial step in placode induction (Thawani et al. 2023) involves the generation of the otic placode (Zine & Fritzsch 2023), which in turn gives rise to the cochlea (ten Donkelaar et al. 2023b). Subsequent transduction of auditory signals within the cochlea relies on specialized sensory hair cells (HCs) and primary auditory neurons within the cochlea. The mechanosensory inner hair cells (IHCs) are organized in a single row and form one-to-one connections with the type I spiral ganglion neurons (SGNs), which relay auditory signals to the brain. Conversely, outer hair cells (OHCs), arranged in three rows, are connected with type II SGNs, which receive input from multiple OHCs. While IHCs serve as primary sensory receptors, OHCs are responsible for cochlear amplification. Most (approximately 95% of ) SGNs are type I SGNs (Dabdoub et al. 2016, Li et al. 2023, Michalski & Petit 2019, Petitpré et al. 2022). The HCs and SGNs are laid out tonotopically, with HCs and SGNs encoding higher frequencies found in the basal region of the cochlea, while those encoding lower frequencies are found in the apical region of the cochlea (Filova et al. 2022b, Hoshino et al. 2021, Yu & Wang 2022). This tonotopic organization is established during development and preserved along the length of the auditory pathway from SGNs projecting to the cochlear nucleus (CN) in the brainstem and continuing via projection pathways to the auditory cortex (AC) (Elliott et al. 2021c, Fritzsch et al. 2019). For a more comprehensive understanding of cochlear anatomy and physiology, the reader should explore recent reviews on the subject (Grothe 2020, Pyott & von Gersdorff 2020).

**Figure 1:**
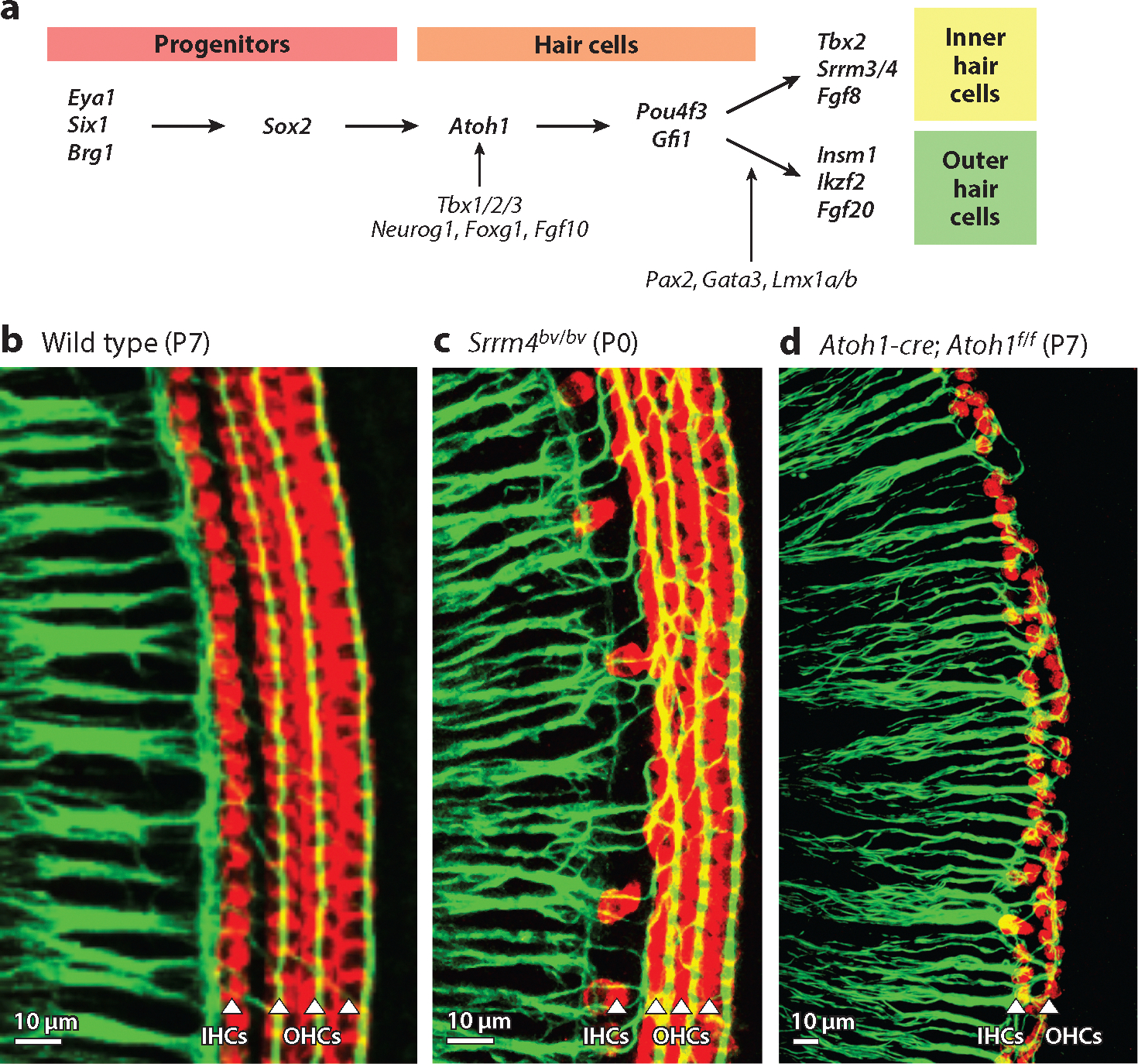
Molecular factors guide the development and organization of the sensory HCs. (*a*) The expression of *Eya1*/*Six1*/*Brg1* and *Sox2* in prosensory progenitors is followed by *Atoh1*, which is needed for hair cell development. The downstream transcription factors *Pou4f3* and *Gfi1* are required to maintain hair cells. *Tbx1/2/3* interact with *Neurog1*, *Foxg1*, and *Fgf10* to regulate neural fate and inner ear morphogenesis. Deletion of these factors results in a reduced number and distribution of SGNs and a shorter cochlea with an increased number of HCs. HCs are lost in mice in the absence of *Pax2*, *Gata3*, and *Lmx1a/b*. *Tbx2*, *Srrm3/4*, and *Fgf8* are needed for the differentiation and viability of IHCs. *Insm1*, *Ikzf2*, and *Fgf20* are necessary for the differentiation of OHCs and/or to form three rows of OHCs. Panel *a* represents concepts and data from Chizhikov et al. (2021), Elliott et al. (2018), Filova et al. (2022b), García-Añoveros et al. (2022), Kaiser et al. (2021), Nakano et al. (2020), and Xu et al. (2021). (*b–d*) The isolated auditory sensory epithelium organ of Corti is immunolabeled with anti-neurofilament (*green*) to label fibers from the SGNs and with anti-Myo7a (*red*) to label HCs. Panels *b–d* adapted with permission from Jahan et al. (2018). (*b*) IHCs and OHCs are organized into one and three parallel rows, respectively, contacted by afferent SGNs. (*c*) The absence of *Srrm4* results in near complete loss of IHCs, with fibers aberrantly targeting the OHCs. (*d*) Delayed expression of *Atoh1* results in one row of IHCs and one row of OHCs that nevertheless remain innervated. Abbreviations: HC, hair cell; IHC, inner hair cell; OHC, outer hair cell; P, postnatal day; SGN, spiral ganglion neuron.

**Figure 2:**
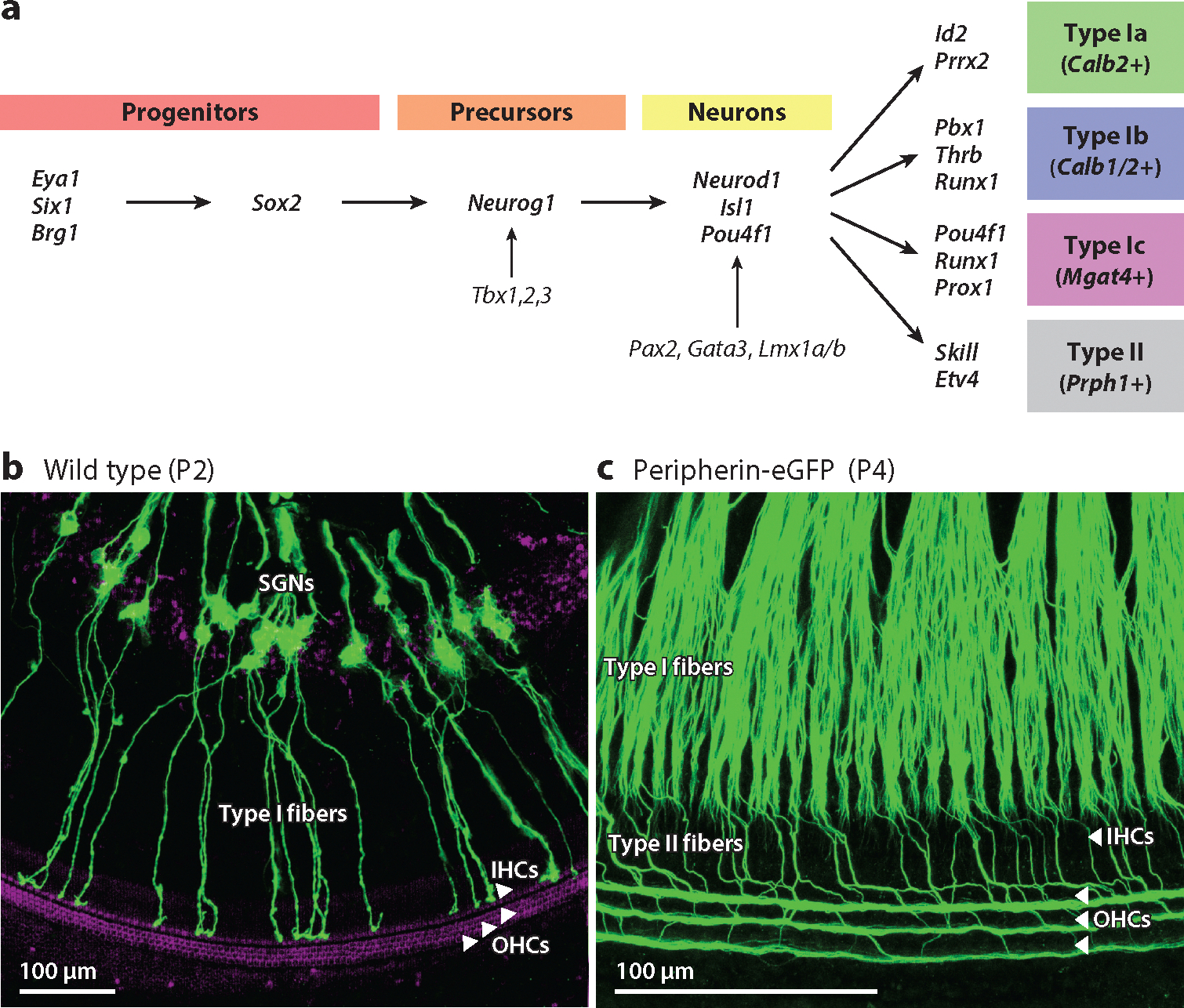
Molecular factors guide the development and organization of the SGNs and their connections. (*a*) Progenitors and precursors depend on the initial expression of *Eya1/Six1/Brg1*, *Sox2*, and *Neurog1*, which activate several genes required for the differentiation of the SGNs. A large set of genes are necessary downstream, but the transcriptional factors *Neurod1*, *Isl1*, and *Pou4f1* are crucial, as well as *Pax2*, *Gata3*, and *Lmx1a/b*. SGNs further differentiate into four populations: type Ia, Ib, Ic, and II. Unique patterns of gene expression are required for the differentiation and identification of these neuronal subtypes. Panel *a* represents concepts and data from Bouchard et al. (2010), Chizhikov et al. (2021), Dvorakova et al. (2020), Elliott et al. (2021a,c), Filova et al. (2022b), Ma et al. (2000), Petitpré et al. (2022), Sun & Liu (2023), and Xu et al. (2021). (*b*,*c*) The isolated organ of Corti is shown, where fibers from the SGNs have been traced with lipophilic dyes (panel *b*, *green*) or identified via transgenic expression of peripherin-eGFP (panel *c*, *green*). Hair cells are identified by immunolabeling with anti-Myo7a (*purple*, panel *b* only). (*b*) By P2, fibers from the SGNs begin to contact the IHCs. Panel *b* adapted from Fritzsch (2023). (*c*) By P4, both IHCs and OHCs are contacted by fibers from the SGNs. Panel *c* adapted from Elliott et al. (2021a) (CC BY 4.0). Abbreviations: eGFP, enhanced green fluorescent protein; IHC, inner hair cell; OHC, outer hair cell; P, postnatal day; SGN, spiral ganglion neuron.

Complex molecular and cellular processes are involved in the development of the cochlea and the formation, differentiation, and maintenance of the HCs and SGNs (Figures 1 and 2). Recurring roles for key transcription factors have been revealed by a combination of reporter-gene studies, in situ hybridization, and immunohistochemistry in combination with gene knockout mice to identify their spatiotemporal contributions (Elliott et al. 2021c, Michalski & Petit 2019, Thawani et al. 2023). Transcription factors will be indicated by gene name, with the protein encoded. As schematized in Figure 1a, initiation of HC progenitors begins with the expression of the transcriptional activators *Eya1/Six1/Brg1*, encoding members of the eyes absent (EYA1) family of proteins, sine oculis-related homeobox 1 (SIX1), and SWI/SNF related, matrix associated, actin dependent regulator of chromatin, subfamily a, member 4 [SMARCA4 (Xu et al. 2021)]. Downstream expression of the transcription factor sex determining region Y (SRY)-box 2 (*Sox2*) is needed to initiate HC differentiation in the inner ear (Dvorakova et al. 2020, Kiernan et al. 2005, Yamoah et al. 2020) and interacts with other genes, including *Wnt*, *Notch*, and *Hes* (Driver & Kelley 2020, Holley et al. 2010, Żak & Daudet 2021). Atonal basic helix–loop–helix (bHLH) transcription factor 1 (*Atoh1*), which encodes a key regulator of neurosensory development in the ear, is required for further differentiation of HCs (Bermingham et al. 1999, Elliott et al. 2021c). Delayed deletion and overexpression of *Atoh1* reduce the survival of HCs (Chonko et al. 2013, Pan et al. 2012, You et al. 2023). Several other genes are needed for the complete development of the HCs (Iyer & Groves 2021, Tang et al. 2023). Downstream genes include various transcriptional regulators (*Pou4f3*, *Gfi1*, *Barhl1*, and others), which are necessary for HC maintenance (Iyer & Groves 2021, Sun & Liu 2023, Tang et al. 2023). Deletion of the transcription factors GATA binding protein 3 (*Gata3*), *Lmx1a/b* (encoding the LIM homeobox transcription factor 1 a/b), T-box 2/3 (*Tbx2/3*), or paired box 2 (*Pax2*) results in the absence of HCs, likely through their interaction with *Atoh1* (Blinkiewicz et al. 2023, Bouchard et al. 2010, Chizhikov et al. 2021, Duncan & Fritzsch 2013, Song & Morrow 2023). An interaction between the bHLH transcription factor neurogenin 1 (*Neurog1*), *Lmx1a*, or *Foxg1* (a member of the fork-head transcription factor family) reduces the expression of *Atoh1*, as the deletion of either results in a shortened cochlea with multiple rows of HCs and supporting cells (Elliott et al. 2021c, Matei et al. 2005). A shorter cochlea and conversions of OHCs into IHCs are associated with deleting the bHLH transcription factor *Neurod1* (Filova et al. 2020, 2022a; Macova et al. 2019).

Various genes are essential in differentiating IHCs and OHCs (Figure 1). OHC development requires downstream expression of the transcriptional repressor insulinoma-associated 1 (*Insm1*) and transcriptional regulator IKAROS family zinc finger 2 [*Ikzf2* (Li et al. 2023, Wiwatpanit et al. 2018)]. Loss of the transcriptional repressor *Tbx2* converts IHCs into OHC-like HCs (García-Añoveros et al. 2022, Kaiser et al. 2022). The combined loss of *Tbx2/3* (Song & Morrow 2023) reduces HCs, while the loss of serine/arginine repetitive matrix 3/4 (*Srrm3/4*) results in IHCs dying at birth (Nakano et al. 2020). The loss of *Bdnf*, which encodes brain-derived neurotrophic factor, results in the loss of apical OHCs, while the loss of the closely related growth factor *Ntf3*, which encodes neurotrophin 3, causes the loss of basal OHCs. The combined loss of *Bdnf* and *Ntf3* in mice results in the loss of all HCs by two months after birth (Kersigo & Fritzsch 2015). In summary, the two types of HCs depend on the initial upregulation of *Atoh1* but subsequently require other factors for proper organization and maintenance (Figure 1b–d).

Like sensory HCs, SGNs are derived from *Eya1/Six1/Brg1-*expressing progenitors (Figure 2a). *Eya1/Six1/Brg1* and *Sox2* define the neurosensory domain of the otocyst (inner ear precursors), whereas *Neurog1* is needed to initiate the proliferation and differentiation of the SGNs (Dvorakova et al. 2020, Kiernan et al. 2005, Ma et al. 2000, Xu et al. 2021, Zine & Fritzsch 2023). A unique trio of transcription factors, *Tbx1/2/3* in concert with *Neurog1*, regulate otocyst processing and otic neurogenesis (Kaiser et al. 2021). The transcriptional repressor *Tbx1* acts as a selector gene that controls neuronal fate in the otocyst (Raft et al. 2004). The combined deletion of both *Tbx2* and *Tbx3* results in the expansion of the otic neurogenic domain and disruption of inner ear morphogenesis. Conditional deletions of *Gata3*, *Lmx1a/b*, and *Dicer* and deletion of *Pax2* result in the complete loss of SGNs (Bouchard et al. 2010, Chizhikov et al. 2021, Duncan & Fritzsch 2013, Kersigo et al. 2011). Deletions of the transcription factors *Neurod1* and *Isl1* or the loss of Schwann cells in *Sox10* or *Erbb2* deletion mutants results in the abnormal migration of SGNs (Filova et al. 2022a,b; Macova et al. 2019; Mao et al. 2014; Morris et al. 2006; Sun & Liu 2023). Downstream neurotrophins are also needed for SGN development and maturation (Rubel & Fritzsch 2002), with the loss of all SGNs occurring in the absence of the neurotrophins encoded by *Bdnf* and *Ntf3* or the genes encoding their receptors, *TrkB* and *TrkC* (Kersigo & Fritzsch 2015). The absence of *Bdnf* alone results in the loss of only about 5% of apical SGNs. In contrast, the deletion of *Ntf3* results in the loss of approximately 95% of SGNs (Elliott et al. 2021b, Fritzsch et al. 2016).

Based on their gene expression profiles, the type I and type II SGNs can be distinguished from one another (Figure 2a). Type I SGNs can be further classified into types Ia–Ic (Petitpré et al. 2018, 2022; Shrestha et al. 2018; Sun et al. 2018). The transcription factor *Runx1* controls SGN subtype differentiation, with the loss of *Runx1* from embryonic SGNs causing more SGNs to adopt Ia rather than Ib or Ic identities (Shrestha et al. 2023). After differentiation, type Ia SGNs are typified by the enriched expression of *Calb2*, which encodes the calcium-binding protein calbindin 2 (CALB2), also known as calretinin, and *Pcdh20*, a member of the protocadherin gene family. Type Ib SGNs show enriched expression of *Calb2* (Siebald et al. 2023). Type Ic SGNs show enriched expression of *Pou4f1*, encoding a neuronal transcription factor; *Lypd1*, encoding a predicted modulator of nicotinic acetylcholine receptors; and *Grm8*, encoding glutamate metabotropic receptor 8. Gene expression profiles also correlate with the targeting of the type I SGNs to the IHCs. CALB2-positive type Ia afferents project preferentially to the pillar face of the IHCs (Petitpré et al. 2022) and thus are suspected to correspond to high–spontaneous rate (SR) fibers, whereas *Lypd2*-positive/CALB2-negative type Ic SGNs project preferentially to the modiolar face of the IHCs and thus are suspected to correspond to low-SR fibers. There is slight variation in the relative abundance of type Ia, Ib, and Ic SGNs across the apical, middle, and basal cochlear turns.

When comparing mammalian HC and SGN development, there is a delay between the differentiation of HCs and SGNs, with SGNs developing basally [embryonic day (E)10.5] to apically (E12.5) and HCs developing apically (E12.5) to basally (E14.5). As seen in older mice in Figure 2b,c, SGN type I fibers first reach IHCs at E13 and OHC type II fibers at E18 (Elliott et al. 2021a). Projections from SGNs first reach the CN at E12 (Schmidt & Fritzsch 2019). The topology between the basal turn (dorsal) and the apical turn (ventral) afferents is established at E14 (Filova et al. 2022b, Krasewicz & Yu 2023). In summary, several genes ensure that the SGNs differentiate into the four types (type Ia–Ic and type II SGNs) and connect the HCs to the CN tonotopically.

## AUDITORY BRAINSTEM AND MIDBRAIN: CONNECTING THE AUDITORY PERIPHERY TO THE BRAIN AND INTEGRATING SIGNALS BINAURALLY

After transduction in the auditory periphery, auditory signals are further processed in structures within the brainstem and midbrain. SGNs send input to neurons in the CN, which is itself divided into several subnuclei, including most notably the anteroventral cochlear nucleus (AVCN), posteroventral CN, and dorsal and ventral cochlear nucleus (DCN and VCN), each with distinct anatomical and functional characteristics important for frequency tuning, intensity coding, and temporal processing (Di Bonito & Studer 2017, Elliott et al. 2021c, Oertel & Cao 2020). The superior olivary complex (SOC) in the brainstem is the next major processing relay. The SOC plays a critical role in binaural hearing and the localization of sound sources in space (Kandler et al. 2020, Kopp-Scheinpflug & Forsythe 2018). A third major relay, via the lateral lemniscus (LL), is the inferior colliculus (IC) (Felmy & Meyer 2020, Malmierca 2015, Rees 2020). Located in the midbrain, the IC receives auditory input from the CN and SOC and is responsible for more complex sound analysis and integration. For a more comprehensive understanding of CN, SOC, LL, and IC anatomy and physiology, the reader is encouraged to explore selected reviews (Elliott et al. 2022, Felmy & Meyer 2020, Kandler et al. 2020, Kopp-Scheinpflug & Linden 2020, Malmierca 2015).

**Figure 3:**
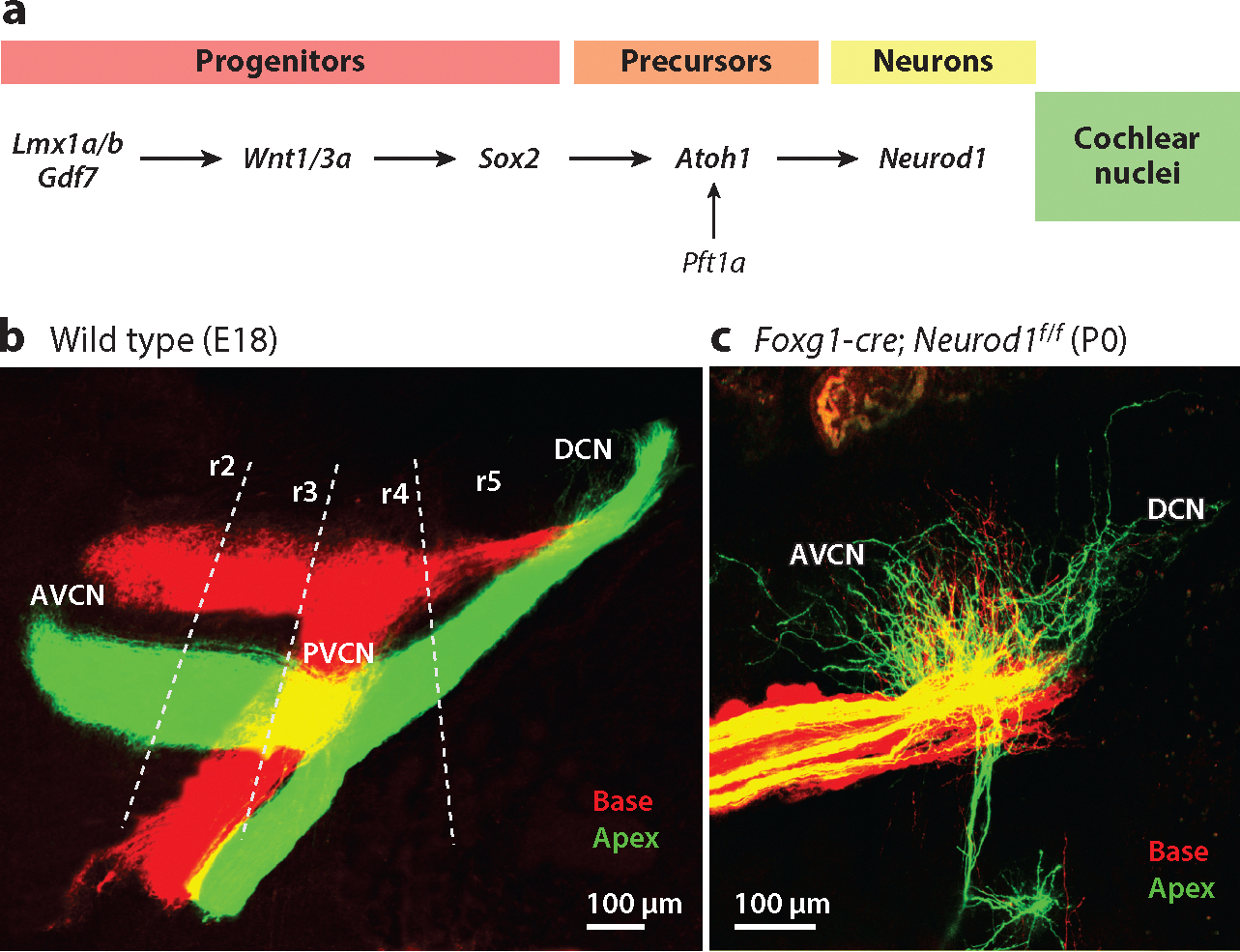
Molecular factors guide the development of projections to the CN and their tonotopic organization. (a) Progenitors depend on *Lmx1a/b*, *Wnt1/3a*, and *Sox2* to express *Atoh1* and then *Neurod1* before they differentiate to develop into excitatory glutamatergic neurons. In addition, *Ptf1a* expression guides the development of inhibitory glycinergic and GABAergic neurons. Panel *a* represents concepts and data from Chizhikov et al. (2021), Elliott et al. (2021c, 2023), Filova et al. (2022a), and Wang et al. (2005). (b) Projection pathways from the inner ear into the CN, including the AVCN, PVCN, and DCN, as well as the VG, are identified using lipophilic dyes. Projections from the cochlear base are labeled by red dye, whereas apical projections are labeled by green dye. The most ventral projections into the CN arise (*green*) from the apical region of the cochlea, while the dorsal projections (*red*) arise from the basal region of the cochlea. (*c*) Deletion of *Neurod1* driven by *Foxg1*-cre results in central projections that fail to segregate tonotopically. Panel *c* adapted with permission from Filova et al. (2022a). Abbreviations: AVCN, anteroventral cochlear nucleus; CN, cochlear nucleus; DCN, dorsal cochlear nucleus; E, embryonic day; P, postnatal day; PVCN, posteroventral cochlear nucleus; r, rhombomere; VG, vestibular ganglion.

The CN is located at the junction between the medulla and pons and originates from rhombomeres 2–5 (r2–5), transient subdivisions within the hindbrain region during early development (Di Bonito & Studer 2017, Elliott et al. 2021c, Farago et al. 2006, Hernandez-Miranda et al. 2017, Nothwang 2016, ten Donkelaar et al. 2023b). The interactions of various genes play a crucial role in organizing neurons within the rhombomeres (Glover & Fritzsch 2022, Schinzel et al. 2021). Upstream of the rhombomeres are *Lmx1a/b* and *Gdf7*, which define the choroid plexus and are needed to express Wnt proteins, which interact with β-catenin, encoded by *Ctnnb1*, for the proliferation of CN neurons (Chizhikov et al. 2021, Elliott et al. 2021c, Rim et al. 2022). Loss of *Lmx1a/b*, *Wnt3a*, or *Atoh1* eliminates all CN neurons (Elliott et al. 2021c, Wang et al. 2005). These developmental factors are schematized in Figure 3a.

Similar to the sensorineural structures in the periphery, neurons in the CN are also arranged tonotopically, with those responding to high frequencies located more dorsally and those responding to low frequencies situated more ventrally (Muniak et al. 2016). Various genes are essential for establishing the tonotopic organization and later cell type differentiation within the CN. The expansion of projections from the CN relies on Ephrin/Eph signaling pathways, which are known to play essential roles in axon guidance and mapping in other sensory systems (Krasewicz & Yu 2023, Milinkeviciute & Cramer 2020) and in maintaining precise tonotopy (Elliott et al. 2023, Hoshino et al. 2021). The tonotopic organization is also influenced by developmental changes in the SGNs and HCs. For example, deletion of *Neurod1* or *Isl1*, which causes alterations in SGN development, results in an overlap of basal and apical projections in the CN (Filova et al. 2020, 2022a,b; Macova et al. 2019) as illustrated in Figure 3b,c. Various findings also highlight the critical but complex role of *Atoh1* in establishing and maintaining the precise tonotopic organization within the CN. Remarkably, the tonotopic organization appears nearly normal after the conditional deletion of *Atoh1* in HCs (Filova et al. 2020) and even after the conditional deletion of *Atoh1* in both HCs and the CN (Elliott et al. 2017). However, conditional deletion of *Atoh1* in the CN by *Hoxb1-cre* or *Egr2-cre*, which deletes expression of *Atoh1* specifically in either r3 and r5 (*Egr2-cre*) or r4 (*Hoxb1*-*cre*), results in subtle tonotopic reorganization within the CN (Di Bonito & Studer 2017, Maricich et al. 2009).

Moreover, more profound caudal projections of site-specific *Atoh1* deletion by *Egr2-cre* correlate with more neurons in the DCN, because neurons in the VCN, which are primarily derived from the *Atoh1*-expressing neuroepithelium (Fujiyama et al. 2009), are lost, whereas neurons in the DCN, which are primarily derived from the *Ptf1a*-expressing neuroepithelium, are spared. Additionally, using *Atoh1-cre* to eliminate *Hox2a/b*, which is only expressed in the AVCN, reduces tonotopic precision and defective sound frequency discrimination in behavioral tests (Karmakar et al. 2017). Several microRNA are needed for normal development in the brainstem (Bordeynik-Cohen et al. 2023).

Several specialized cell types can be identified in the CN, with each cell type contributing to processing various aspects of auditory information, including sound intensity, timing, frequency, and spatial localization (Kopp-Scheinpflug & Linden 2020). The major cell types include the excitatory principal neurons, of which the bushy cells, stellate cells, and octopus cells carry information out of the VCN, and the fusiform and giant cells, which carry information out of the DCN (Malmierca 2015, Oertel & Cao 2020). The main inhibitory interneurons include the cartwheel cells, which modulate neuronal activity within the CN (Oertel & Cao 2020, Trussell & Oertel 2018). Various gene networks differentiate the various cell types within the CN. The earliest CN neurons proliferate between approximately E10 and E14. These neurons are derived from rostral r2–5 from *Atoh1*-positive and *Ptf1a*-positive neuroepithelial domains (Elliott et al. 2023, Fujiyama et al. 2009, Iskusnykh et al. 2016, Wang et al. 2005). All excitatory, glutamatergic neurons in the CN derive from the *Atoh1* domain. Neurons that derive from the *Ptf1a* domain are either glycinergic or GABAergic. The *Ptf1a* domain is larger in r4–5 than in r2–3 (Figures 2 and 3), which correlates with the fewer inhibitory neurons in the VCN (Fujiyama et al. 2009). Large and small spherical bushy cells that form a large, calyx-shaped afferent terminal arise from r2. In contrast to spherical bushy cells, globular bushy cells interact with prominent gaps in the calyx and originate primarily from r3 (Maricich et al. 2009, Oertel & Cao 2020). D-, T-, and likely also L-stellate cells originate from r3 (Ngodup et al. 2020) and are either glutamatergic (T-stellate) or glycinergic (D-stellate). R4 populations give rise to the octopus (Lu et al. 2022) and giant cells, dependent on *Atoh1* expression. R5 populations give rise to *Atoh1*-expressing neurons, including unipolar brush cells (UBCs), fusiform cells, and granule cells, and a large, additional subpopulation of *Ptf1a*^+^-derived neurons give rise to the GABAergic Golgi cells and superficial stellate cells as well as glycinergic cartwheel and tuberculoventral cells (Trussell & Oertel 2018).

SGNs synapse with many different cell types within the CN (Di Bonito & Studer 2017). Type I SGNs connect all *Atoh1*-derived neurons in the CN by end bulbs or terminating small connections. The type I and type II fibers project to the CN in parallel, but type II fibers only reach the UBCs and granule cells (Muniak et al. 2016, Trussell & Oertel 2018). Various genes have been implicated in differentiating specific cell types in the CN. For example, deleting *bHLHb5*, which encodes a transcription factor belonging to the bHLH family, eliminates two types of DCN neurons: UBCs and cartwheel cells (Cai et al. 2016). The expression of *Lbx1*, a homeobox gene, is observed in stellate and cartwheel cells of the DCN but not in Golgi cells, indicating that different genetic programs define cell types originating from the *Ptf1a* lineage (Elliott et al. 2023, Schinzel et al. 2021).

The SOC receives auditory information from the CN, a major convergence point for binaural auditory information. Cells of the SOC are generated from E10.5 to E14.5 in mice. Additional cells are generated later at E17.5 in the CN, from where they migrate to the SOC and LL (Di Bonito et al. 2017). Neurons are, in part, dependent on *Atoh1* but also on the homeobox-containing gene *En1* (Milinkeviciute & Cramer 2020). Many SOC neurons are generated in r4 (Di Bonito & Studer 2017). Conditional deletion of *Atoh1* by *Egr2-cre* from r3–5 reduces the size of the SOC (Maricich et al. 2009); however, in older mice, it is unclear how many SOC neurons are primarily versus secondarily lost as a consequence of CN disruption. In summary, two classes of neurons in the CN are identified by their dependence on *Atoh1* and *Ptf1a*. Neuronal proliferation requires the upstream expression of *Lmx1a/b*, *Gdf7*, various *Wnt* genes, *Ctnnb1*, and the downstream expression of large gene sets to further differentiate neuronal types, including *Pou4f1*, *Barhl1*, and *Lhx2/9* for *Atoh1*-dependent neurons and *Foxd3*, *Foxp2*, *Lbx1*, *Pax2*, and *Lhx1/5* for *Ptf1a*-dependent neurons.

**Figure 4:**
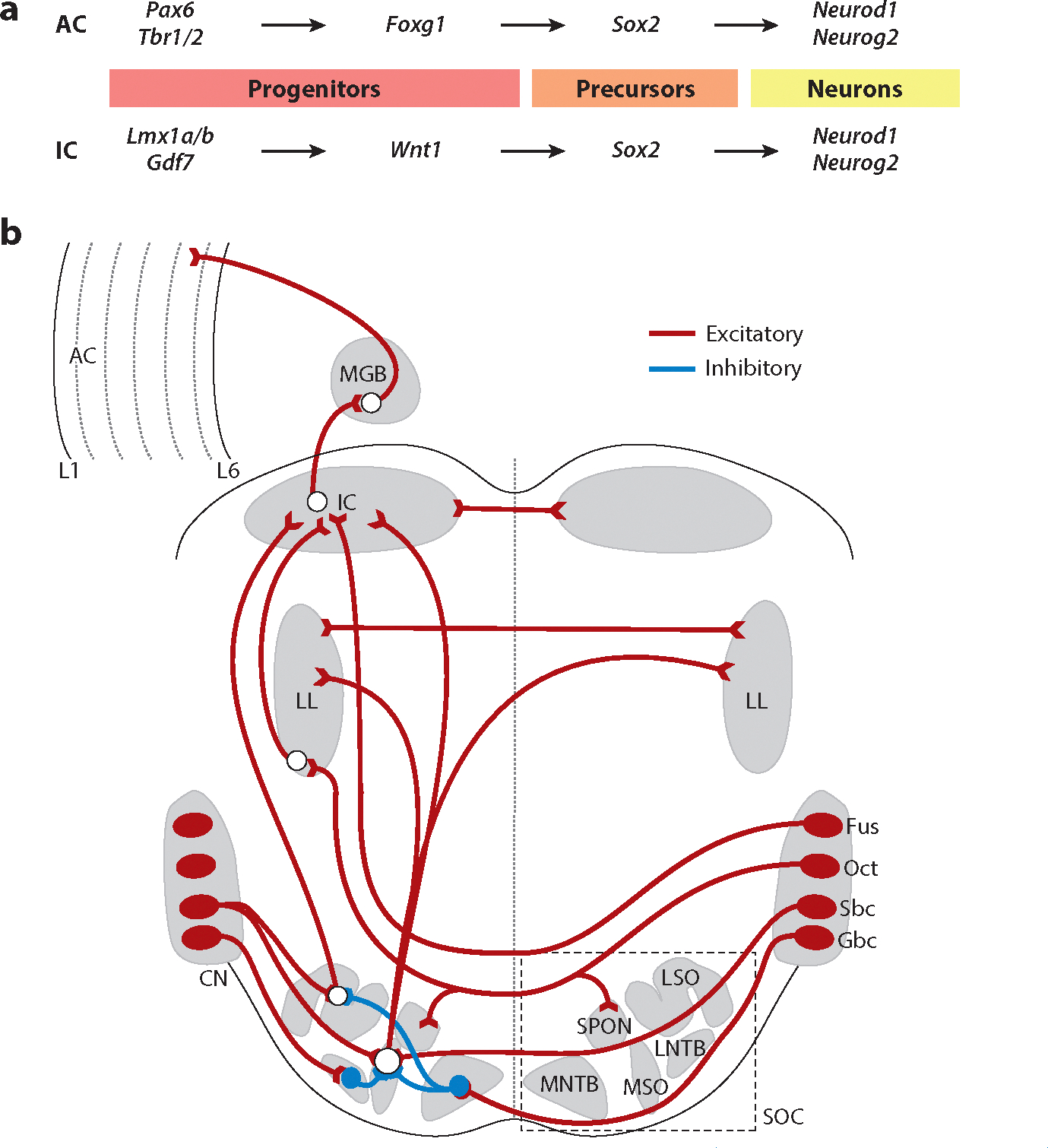
Molecular factors guide the development of neurons in the IC and AC and establish the projection pathways from the brainstem to the AC. (*a*) Progenitors in the AC depend on *Pax6*/*Tbr1/2* and *Foxg1* to induce *Sox2* to in turn induce neurons that depend on *Neurod1* and *Neurog2* to differentiate into excitatory glutamatergic neurons. Progenitors in the IC depend on *Lmx1a/b/Gdf7*, *Wnt1*, and *Sox2* to induce neurons that also then depend on *Neurod1* and *Neurog2* to differentiate into excitatory glutamatergic neurons. Panel *a* represents concepts and data from Glover et al. (2018) and Hevner (2022). (*b*) Outputs of the four major types of neurons in the CN project to the SOC, which comprises four main nuclei: the LSO, MSO, MNTB, and LNTB. The LSO receives a binaural input originating from the ipsilateral Sbcs and contralateral Gbcs, the latter of which are inhibited by neurons from the MNTB. The MSO receives bilateral excitatory input from the Sbcs and bilateral inhibitory input from the Gbcs via the MNTB and the LNTB. The Octs provide bilateral input to the SPON and eventually connect with the LL. Not shown are the T-stellate cells, which terminate in distinct regions of the SOC, LL, and IC. The output from the dorsal CN, from Fus neurons, bypasses the SOC to reach the contralateral IC. Additionally, the MGB receives input from the IC and projects to the AC. These interactions play significant roles in processing auditory information along the neural pathway. The molecular factors guiding the development of these projection pathways are poorly understood. Panel *b* represents concepts and data from Kandler et al. (2020), Malmierca (2015), Molnár et al. (2020), Pecka & Encke (2020), Rees (2020), and Tran et al. (2023). Abbreviations: AC, auditory cortex; CN, cochlear nucleus; Fus, fusiform; Gbc, globular bushy cell; IC, inferior colliculus; L, layer; LL, lateral lemniscus; LNTB, lateral nucleus of the trapezoid body; LSO, lateral superior olive; MGB, medial geniculate body; MNTB, medial nucleus of the trapezoid body; MSO, medial superior olive; Oct, octopus cell; Sbc, spherical bushy cell; SOC, superior olivary complex; SPON, superior paraolivary nucleus.

The IC serves as a key integration center for auditory input. It receives input from the CN, SOC, and LL and directs output to the thalamus’s medial geniculate body (MGB; covered in the next section). The reader is again referred to several references for more information about the anatomy and physiology of the IC (Malmierca 2015, Rees 2020). Various genes play crucial roles in developing the IC and related structures, from their development to volume and cell type expression (Figure 4a). The gene *Rab23* (a member of the Ras oncogenes) encodes a small GTPase of the Ras superfamily that antagonizes the sonic hedgehog (*Shh*) signaling molecule known to be critical in the developmental patterning of the IC (Eggenschwiler et al. 2006). The complete loss of *Wnt1*, which encodes a key regulator of cell fate, proliferation, and patterning (Nusse & Clevers 2017) and acts downstream of the transcription factor *Lmx1b*, eliminates the entire midbrain (Glover et al. 2018, Mastick et al. 1996). The deletions of *Fgf8*, which encodes a fibroblast growth factor, and the transcription factors *Pax*2 and developing brain homeobox 1 (*Dbx1*), among others, reduce the size of the IC (Driscoll & Tadi 2022, Tran et al. 2023, Urbánek et al. 1997, Watson et al. 2017).

Conversely, conditional activation of the Shh receptor Smoothened (*Smo*) using *Pax2-cre* results in exuberant development of the IC and the cerebellum ( Jahan et al. 2021). Misexpression of *Isl1* under *Pax2* regulatory sequences reduces the size and disrupts the function of the IC (Chumak et al. 2021). *Pax3*, *Pax7*, and Meis homeobox 2 (*Meis2*) are expressed downstream of *Pax2* and affect the development of the IC roof plate (Agoston et al. 2012, Nakamura 2020). Orthodenticle homeobox 2 (*Otx2*) is driven by the expression of *Sox2*, *Ascl1*, *Neurod1*, *Neurog2*, and *Dll3* and shapes early initiation and differentiation of the IC (Henke et al. 2009). *Neurod1* is expressed downstream of *Neurog1* and *Neurog2* in the IC (Gurung & Fritzsch 2004, Miyata et al. 1999). The embryonic patterning gene *Dbx1* is required for postnatal neuronal survival in the IC (Tran et al. 2023). Beyond this early regulation, subsequent expression of glutamatergic, glycinergic, and GABAergic neuronal phenotypes in the IC depends on various neurotrophins for survival (Rees 2020). In summary, neuronal proliferation and differentiation in the IC depend on a large set of interacting genes. Much work remains to be done to understand the molecular developmental factors that are essential for adequately organizing the neuronal connections between the CN and the IC.

## THALAMUS AND CORTEX: CONNECTING THE BRAINSTEM AND MIDBRAIN TO THE CORTEX TO INTEGRATE AND PERCEIVE AUDITORY SIGNALS

The MGB is the relay nucleus located within the thalamus that receives auditory input from the IC and transmits it to the AC (Budinger 2020), which is located in the cerebral cortex and is essential for sound perception (Malone et al. 2020, Molnár et al. 2020). The MGB and AC are both tonotopically organized but in more complex arrangements than seen in the periphery and other brain regions (Polley & Takesian 2022, Polley et al. 2007). The reader is referred to a few reviews for more information on the anatomical organization and physiology of the MGB (Kanold et al. 2014, Luhmann et al. 2022) and AC (Rauschecker 2020, Rolls et al. 2023).

Similar to rhombomeres in the hindbrain, the forebrain is divided into transient developmental segments called prosomeres, numbered rostrally to caudally and characterized by specific molecular and genetic markers that determine patterning and differentiation. The diencephalon, which includes the thalamus, hypothalamus, epithalamus, and subthalamus, originates from prosomere 2 (p2) and is defined by specific gene expression patterns (Puelles 2019, Puelles et al. 2012, ten Donkelaar et al. 2023b). P2 is caudal to the neuropore (Fritzsch & Martin 2022) and is separated from the zona limitans (Puelles et al. 2015), which delineates the expression of *Foxg1* in p3 from the adjacent expression of *Foxd1* in the ventral part of p2 (Newman et al. 2018, Puelles et al. 2015). *Pax6* is essential for the development of the forebrain and thalamus (Manuel et al. 2022). In rats, the MGB is generated between E13 and E15 and follows a chrono-architectural development (Altman & Bayer 1989). In mice, MGB development starts before auditory sensory perceptions begin, with connections from the IC arriving between E13 and E18 (Gurung & Fritzsch 2004). Genes expressed early in the developing MGB include *Wnt3*, *Tcf4*, *Meis2*, and *Irx3* (Puelles et al. 2015) as well as *Zic4* and *Foxp2* (Horng et al. 2009, Scharff & Petri 2011). Moreover, *Gbx2* is necessary to define the boundaries of the MGB (Glover et al. 2018). In addition, several downstream genes, including several bHLH genes (*Neurog2*, *Ascl1*, *Olig2*, and *Neurod1*), that are needed for the normal development of the MGB have been identified. Further analysis of conditional deletions is needed to clarify their interactions (Dennis et al. 2019). Finally, T-box brain transcription factor 1 (*Tbr1*) is expressed early and defines glutamatergic forebrain neurons. Without glutamate, neurons of the MGB fail to develop projections to the forebrain (Hevner et al. 2001). Alterations in connectivity result from the targeted deletion of other crucial genes, including *Pax6*, *Foxp2*, *Wnt3*, *Tcf4*, and *Irx3*, but they have not been thoroughly described and require further investigation.

The AC receives input from, and reciprocally provides feedback to, the MGB. Surrounding the primary auditory cortex (A1) are additional cortical areas [approximately 6 in mice and up to 30 in humans (Budinger & Kanold 2018)] that have specific roles in processing auditory information, are interconnected with A1, and also exhibit a gradient of tonotopic input (Polley & Takesian 2022, Polley et al. 2007). In addition to these cortical areas, the AC displays a columnar organization with six distinct layers that are 1.1–1.2-mm thick and contain neurons, astrocytes, and glia (Budinger 2020, Molnár et al. 2020). Layer I is adjacent to the pia, layer II contains small neurons, layer III has a mix of pyramidal and nonpyramidal neurons, layer IV comprises smaller neurons, layer V is the thickest layer and contains larger pyramidal neurons, and, finally, layer VI has a combination of pyramidal and nonpyramidal neurons (Budinger 2020, Malmierca 2015). Neurons of the AC are diverse and include spiny stellate, multipolar, chandelier, bi-tufted, double-bouquet, bipolar, and horizontal basket neurons and the superficial Cajal-Retzius neurons that interact with Martinotti cells from layer V (Budinger 2020). The pyramidal neurons are glutamatergic excitatory projecting neurons, whereas the inhibitory interneurons are GABAergic and glycinergic (Molnár et al. 2020, Mukherjee et al. 2022).

During early development, crucial gene interactions guide the various complex processes, including proliferation, migration, and differentiation, that form the cortex—including the AC—from the forebrain. Gene expression during forebrain development is well documented and involves key transcription factors and signaling molecules, including *Pax6*, *Tbr1*, *Tbr2*, *Otx1*, *Shh*, empty spiracles homeobox 2 (*Emx2*), and *Foxg1* (Englund et al. 2005, Kral & Pallas 2011), before neural tube closure of the neuropore (Fritzsch & Martin 2022, ten Donkelaar et al. 2023b). About 80% of neurons develop into primary neurons, while the remaining 20% develop into interneurons (Hevner 2022, Luhmann et al. 2022, Molnár et al. 2020). Cortical inhibitory interneurons are generated from the ganglionic eminences and migrate into the cortex. In addition, excitatory neurons are generated locally and migrate from the ventricular layer progenitor cells (Molnár et al. 2020, ten Donkelaar et al. 2023a, Yamoah et al. 2023). A specific set of genes, including *Pax6*, *Tbr1*, *Tbr2*, *Dlx1*–*3*, *Nkx2.1*, and others, are essential for normal forebrain development (Arnold et al. 2008, Englund et al. 2005, Hevner 2022, Manuel et al. 2022, Puelles et al. 2015, ten Donkelaar et al. 2023b). There are precise and reproducible spatiotemporal patterns of gene regulation during forebrain development. This regional activation plays a critical role in producing regionally specific cell types. For example, *Pax6* deletion alters the expression of various genes, including *Ascl1*, *Neurog2*, *Neurod1*, *Tbr1*, *Sox5*, *Sox9*, and *Hes5*, while the expression of *Foxg1*, for example, remains relatively normal (Manuel et al. 2022). *Pax6* and *Foxg1* both depend on *Shh* and various bone morphogenetic proteins, which play an established and critical role in adult neurogenesis and gliogenesis throughout the mammalian brain and interact in an opposing manner to regulate the competence of cortical cells (Manuel et al. 2010, 2022). Double deletion of *Pax6* and *Foxg1* abolishes the expression of various genes, including *Ascl1*, *Olig2*, *Gsx2*, and *Dlx1*–*3*, leading to redirected neuronal variations (Manuel et al. 2022). *Neurod1* is expressed downstream of *Neurog2* and plays a vital role in the differentiation of cortical neurons, paralleling its established role in differentiating granule neurons in the hippocampus (Dennis et al. 2019, Liu et al. 2000, Miyata et al. 1999). The exact number of interneurons developing after the reduction and loss of *Neurod1* remains unclear (Mukherjee et al. 2022). *Foxg1*-*cre*-mediated deletion of *Dicer1*, encoding the enzyme responsible for forming active microRNAs, results in the loss of most neurons (Kersigo et al. 2011). An extensive set of microRNAs are required for forebrain development and have been identified but remain incompletely characterized (Park et al. 2022, Piwecka et al. 2017). More work is necessary to extend this inventory and identify their contributions. The various genes involved in the developmental organization of the AC are shown in Figure 4a.

In humans, the process of cortical neurogenesis begins around the sixth week of gestation and continues throughout fetal development (Molnár et al. 2020). In mice, AC neurons are generated between E11.5 and E13.5. The earliest neurons to develop are Cajal-Retzius neurons, appearing slightly earlier, around E10.5–12.5 (Dennis et al. 2019). Neuronal migration contributes to the formation of different cortical layers (Molnár et al. 2020). Two regions, the marginal and subplate zones, are present in the preplate during cortical development (Kanold & Luhmann 2010). The earliest neurons to emerge in the cerebral cortex are subplate neurons, which establish a transient circuitry connecting the auditory thalamus and cortical layer 4. These neurons undergo a process of elimination during the onset of hearing. The specific subplate neurons that survive eventually contribute to the formation of cortical layer 6 (Goodrich & Kanold 2020, Luhmann et al. 2022, Yamoah et al. 2023). These surviving subplate neurons may also contribute to the development of altered circuits that are associated with certain neurological disorders (Molnár et al. 2020). The maturation of input from cortical neurons exhibits a delay in somatosensory, visual, and auditory cortices, which then undergo functional reorganization to form oriented columns. Excitatory and inhibitory circuits are also defined in the developing AC, with predominantly AMPA- and NDMA-mediated glutamatergic input to the subplate and layer 4 with local GABAergic activity (Kanold & Luhmann 2010). Progressive segregation leads to discrete input/output relationships between the MGB and AC (Gurung & Fritzsch 2004). The dynamic processes of development and connectivity in the AC play a crucial role in auditory processing and perception, which change the connections between the MGB and AC (Mukherjee et al. 2022).

In summary, specific genes are needed for general forebrain development and connectivity (Figure 4b). Specific sets of genes unique to the AC have not yet been fully identified (Hevner 2022, ten Donkelaar et al. 2023a). There is, therefore, still much to learn about the underlying genetic mechanisms that lead to normal AC development (Molnár et al. 2020) and circuitry (Cossart & Garel 2022).

## CONCLUDING REMARKS

In this review, we have presented a comprehensive and integrative overview of the critical genes involved in auditory pathway development, shedding light on the molecular and genetic mechanisms that underlie the intricate process of forming the auditory system. Through the exploration of recent studies, we highlighted the pivotal, reoccurring, and interacting roles of genes such as *Eya1*, *Sox2*, *Atoh1*, *Neurog1*, *Otx1*, *Lmx1a/b*, *Pax6*, *Shh*, *Emx2*, *Foxg1*, *Tbr1*, and *Tbr2* in guiding the development, differentiation, and organization of auditory structures from the inner ear to the AC.

A deeper understanding of the complexity of auditory development holds great promise for potential therapeutic advancements in auditory-related disorders. These insights have wide-ranging applications, advancing strategies for early diagnosis, biomarker monitoring, and personalized medicine treatment approaches and guiding the development of targeted gene and pharmaceutical therapies to prevent or decelerate the progression of auditory disorders and promote repair and regeneration of auditory structures. These insights will benefit individuals with congenital deafness (Michalski & Petit 2022) and inform strategies to treat acquired forms of hearing loss, including age-related hearing loss (Elliott et al. 2022). To realize this potential, applying advanced tools and techniques such as single-cell transcriptomics and CRISPR-Cas9 gene editing (Park et al. 2022) will be crucial in driving future research and clinical interventions.

Despite significant progress in understanding the development of the auditory pathway, many crucial questions remain unanswered (Luhmann et al. 2022). The precise roles and interactions of numerous genes within specific auditory substructures remain to be fully elucidated, necessitating focused investigations employing established genetic models. Exploring the interplay between genetic and environmental determinants of auditory function, including the impact of environmental factors and sensory experience on gene expression during auditory development, remains a much-needed area of research. Finally, our understanding of how gene regulatory networks impact complex auditory processing involving the interaction of the auditory and nonauditory structures remains incomplete. These interactions are essential for speech and language development and musical appreciation. The application of advanced in vivo physiological approaches (Cossart & Khazipov 2022), whole-brain imaging (Patisaul 2020), and behavioral techniques in animal models (Arac et al. 2019) will be essential for answering these questions.
